# Reach, Engagement, and Retention in an Internet-Based Weight Loss Program in a Multi-Site Randomized Controlled Trial

**DOI:** 10.2196/jmir.9.2.e11

**Published:** 2007-05-09

**Authors:** Russell E Glasgow, Candace C Nelson, Kathleen A Kearney, Robert Reid, Debra P Ritzwoller, Victor J Strecher, Mick P Couper, Beverly Green, Kevin Wildenhaus

**Affiliations:** ^5^HealthMediaIncAnn ArborMIUSA; ^4^University of MichiganAnn ArborMIUSA; ^3^Group Health CooperativeSeattleWAUSA; ^2^Kaiser Permanente Care Management InstituteOaklandCAUSA; ^1^Kaiser Permanente ColoradoDenverCOUSA

**Keywords:** Internet, weight loss, recruitment, representativeness, retention, attrition, adherence, behavior change, randomized controlled trial, consumer health informatics

## Abstract

**Background:**

Research increasingly supports the conclusion that well-designed programs delivered over the Internet can produce significant weight loss compared to randomized controlled conditions. Much less is known about four important issues addressed in this study: (1) which recruitment methods produce higher eHealth participation rates, (2) which patient characteristics are related to enrollment, (3) which characteristics are related to level of user engagement in the program, and (4) which characteristics are related to continued participation in project assessments.

**Methods:**

We recruited overweight members of three health maintenance organizations (HMOs) to participate in an entirely Internet-mediated weight loss program developed by HealthMedia, Inc. Two different recruitment methods were used: personal letters from prevention directors in each HMO, and general notices in member newsletters. The personal letters were sent to members diagnosed with diabetes or heart disease and, in one HMO, to a general membership sample in a particular geographic location. Data were collected in the context of a 2×2 randomized controlled trial, with participants assigned to receive or not receive a goal setting intervention and a nutrition education intervention in addition to the basic program.

**Results:**

A total of 2311 members enrolled. Bivariate analyses on aggregate data revealed that personalized mailings produced higher enrollment rates than member newsletters and that members with diabetes or heart disease were more likely to enroll than those without these diagnoses. In addition, males, those over age 60, smokers, and those estimated to have higher medical expenses were less likely to enroll (all *P* < .001). Males and those in the combined intervention were less likely to engage initially, or to continue to be engaged with their Web program, than other participants. In terms of retention, multiple logistic regressions revealed that enrollees under age 60 (*P* < .001) and those with higher baseline self-efficacy were less likely to participate in the 12-month follow-up (*P* = .03), but with these exceptions, those participating were very similar to those not participating in the follow-up.

**Conclusions:**

A single personalized mailing increases enrollment in Internet-based weight loss. eHealth programs offer great potential for recruiting large numbers of participants, but they may not reach those at highest risk. Patient characteristics related to each of these important factors may be different, and more comprehensive analyses of determinants of enrollment, engagement, and retention in eHealth programs are needed.

## Introduction

There have been recent encouraging reports about the efficacy of Internet-based weight loss interventions [1-3], but many questions remain about the appeal and applicability of eHealth programs in real-world settings [4-6]. Specifically, it appears that computer-assisted and Internet programs may be effective for reducing fat intake [7] and increasing weight loss when supplemented by electronic health coaches [2,8,9].

One of the issues in need of greater understanding is recruitment to and participation in Internet-based health promotion programs [10]. We need to understand both the characteristics and motivation of those who participate versus those who decline, as well as the effects of different types of recruitment strategies. There has been considerable work on recruitment methods for clinical trials with in-person visits, especially on recruiting minority participants [11,12], but there is little controlled research on recruitment to Internet-based programs [13]. The ability to efficiently recruit and serve large numbers of consumers in remote locations is one of the key potential advantages of Internet-based programs, but better documentation and detailed understanding of this issue are needed.

An emerging issue of concern for Internet-based health behavior change interventions is high levels of attrition [1,14-16]. Participants use Internet-based resources differently than they do other modalities such as group and in-person programs, and for Internet programs serving large numbers of participants that do not heavily screen users or conduct studies under efficacy conditions, it has proven difficult to obtain follow-up information on a high percentage of initial participants [1,14-16]. At present, it is unclear what patient characteristics are related to active engagement with Internet program content and to participation in follow-up assessments and whether these characteristics are similar or different.

Based on previous promising work, a tailored online behavioral weight loss program (HealthMedia’s Balance) was evaluated in a randomized controlled trial with and without the addition of extended goal setting and feedback and more intensive nutritional information. A prior multi-site randomized study of 2862 participants found the basic Balance program to produce significantly greater weight loss than the control condition [1].

The purposes of this paper are to (1) report on enrollment rates for an innovative, large-scale, Internet-based weight loss study, (2) analyze levels of program engagement and retention at 12-month follow-up, and (3) investigate recruitment method, setting, and patient characteristics associated with enrollment, program engagement, and retention in follow-up assessment.

## Methods

### Settings and Recruitment

The data reported here are part of a larger study assessing the impact of adding a nutrition component, a goal-setting component, or both to a tailored online weight management program (HealthMedia's Balance). The study was conducted in three prepaid group practice HMOs—the Ohio and Colorado regions of Kaiser Permanente, and Group Health Cooperative with enrollees from Washington state and Idaho. Health plan members with and without chronic illnesses (diabetes and coronary artery disease) were invited to participate, either through personal letters from medical leaders or through notices in general member communications such as HMO newsletters, flyers, and posters. Recruitment began in March 2004 and continued through early December 2004. Approval was obtained from Institutional Review Boards in each health plan. Interested members were directed to a study website where they completed a baseline assessment, reported their height and weight, learned whether they met the study criteria, and gave informed consent. Potential participants were excluded from the study if they had a body mass index (BMI) below the study minimum (< 30 for general membership and < 25 for those with chronic illness), were using drugs or surgery for weight loss, were participating in another organized weight loss program, or were physically or medically unable to exercise. Members excluded for these reasons were offered the opportunity to receive the basic Balance program if they wished. Respondents were also told that the Balance program was not intended for pregnant women, for those with eating disorders, or for those who had been diagnosed with heart failure; members who indicated that any of these categories applied to them were excluded.

Participants who met the enrollment criteria and consented to the study were randomly assigned within HMO to one of four interventions composed of combinations of three HealthMedia programs: (1) the 6-week Balance weight loss program alone; (2) Balance plus the 8-week nutrition management module Nourish; (3) Balance plus a simultaneous goal-setting component called Achieve; or (4) Balance plus Nourish and Achieve. Emails asking participants to complete follow-up surveys were sent to all participants 3, 12, and 18 months after enrollment. Participants were informed at the outset of the study that they would receive a US $10 gift certificate from Amazon.com or a similar online vendor each time they completed a follow-up questionnaire. Although not the purpose of this paper, for context we note that participants in all interventions were successful at losing weight.

Because of the low participation rate in follow-up assessments, a sample of nonrespondents to the 12-month online survey who provided mailing addresses were sent a printed survey by mail, along with US $10 cash. Of the 1796 nonrespondents, 913 were sent a mail survey; of these, 586 returned the completed survey, for a 64% response rate to the mail follow-up. (This compares to 56% for a mailed follow-up and 59% for a telephone follow-up in the earlier evaluation of the Balance program [15].) This follow-up effort boosted the unweighted 12-month response rate from 22.3% to 47.6% and the weighted rate to 72.7%. The latter rate is estimated based on the assumption that if we sent the mail follow-up to all nonrespondents to the 12-month online survey, they would respond at the same rate as the randomly selected subsample. Results from this additional mail assessment are described in Couper et al [15] and did not differ from data collected via the Internet or alter the results reported below [15].

### Recruitment Methods

Two target populations were included in the study, and different recruitment approaches were used for each. The populations of interest were (1) overweight health plan members generally and (2) overweight members with chronic illnesses for which weight management is a key part of treatment. The recruitment approaches were personal letters of invitation mailed to members’ homes, for those with diabetes or coronary artery disease (CAD), and announcements about the study in mailed member newsletters and flyers posted at HMO facilities, for general members. An exception to this, which allowed a direct experimental comparison, is that in one HMO a sample of general adult members in one geographic region and a sample of members with hyperlipidemia also received personalized letters.

#### Personal Letters

Personal letters of invitation were sent to randomly sampled members in diabetes registries at all three health plans and in CAD registries at two plans. Letters were also sent to randomly selected overweight members of HMO 1 in a hyperlipidemia registry with no known CAD and to a random sample of the HMO 1 general adult membership. Since only members with BMI ≥ 30 (≥ 25 for those with diabetes or CAD) were eligible to participate in the study, BMI values recorded in the electronic medical record were examined for the two years prior to the beginning of study recruitment. Members whose most recently recorded BMI during this period was lower than the appropriate cutoff point were excluded from the sample. Members who had no BMI recorded during the two-year period were retained in the sample. Only members aged 18 years and over were considered. Table 1 gives the numbers of letters sent, by population and health plan.

The three HMOs employ consistent criteria for registry membership, using both the International Classification of Diseases, Ninth Revision (ICD-9) codes and specific pharmaceutical dispenses to identify patients. Patients in the diabetes registries were identified using either ICD-9 codes, specific pharmaceutical dispenses, or laboratory data indicating diabetes (two fasting blood sugars over 126 mg/dL or two random blood sugars over 200 mg/dL in the prior 12 months). Patients in the hyperlipidemia registries were identified by pharmaceutical dispenses and laboratory criteria. The two HMOs using CAD registries used the following criteria to identify patients with CAD. Patients had to have at least one of the following: (1) a hospital discharge (alive) with a principal or secondary diagnosis of acute myocardial infarction (AMI), percutaneous transluminal coronary angioplasty (PTCA), or coronary artery bypass graft (CABG), (2) a hospital discharge (alive) with a principal diagnosis of other acute or subacute ischemic heart disease, or (3) three or more outpatient visits with a diagnosis (principal or secondary) of CAD within a 36-month period.

The letters sent to potential participants were signed by physician leaders from the appropriate health plan. The letters gave instructions and pass codes for accessing the study website and a telephone contact at their plan. The letters described the study as an evaluation of online programs for helping people to lose weight and invited the addressee to participate in the study “if you are one of the thousands of people who say they want to lose weight.” Members were told that to be eligible they must be overweight, a current member of the health plan, and have an email address and the ability to access the Internet at least once or twice a week.

#### Newsletters and Flyers

In HMOs 2 and 3, recruiting from the general membership was done primarily through general announcements in the quarterly member newsletters, although flyers describing the study were also posted and/or distributed at some local facilities. The newsletter announcements included a brief description of the study, gave plan-specific pass codes and instructions, and provided the name and telephone number of a local contact person. The newsletters were sent to all plan members in the region. Approximately 293000 and 121000 newsletters were distributed in the two regions, respectively. Based on state Behavioral Risk Factor Survey (BRFS) data on obesity rates, we estimate that 46827 and 30119 eligible adults received the newsletters, respectively, in HMO 2 and 3.

### Program Descriptions

The HealthMedia Balance program has been described in detail elsewhere [1]. Briefly, it is a 6-week online self-help program that uses data from a baseline assessment to create an individually tailored weight management plan. Dimensions on which the program is tailored include health and medical history, prior weight loss efforts, intrinsic and extrinsic motivators for managing weight, perceived barriers to change, attitudes and stereotypes about overweight people and weight loss, social support systems, body image, nutritional habits, and physical activity. The program does not advocate a specific diet. Participants receive an initial program guide followed by newsletters containing tailored action plans 1 week, 3 weeks, and 6 weeks after enrollment.

While the Balance weight management program is the primary intervention in this study, we wanted to look at the potential impact of adding two additional Web-based, tailored interventions: Achieve and Nourish. Achieve is a goal-setting program delivered simultaneously with Balance that uses participant-reported performance data (ie, attributions for previous failure, motivation for continued performance, self-efficacy for continued performance) to determine follow-up questions and subsequent goals. The user may then adjust any goal according to his or her preference. Nourish is an online nutrition program that is very consistent with the Balance program. Nourish uses the same format for collecting information via an initial questionnaire to tailor nutrition advice to the specific needs and interests of the user. It consists of a guide, three sequential tailored newsletters, and email notifications delivered over an 8-week period. In this study, subjects received the Nourish program after first completing the Balance program.

### Measures

This paper examines enrollment numbers and rates, engagement rates, and 12-month retention rates for the different recruitment approaches, target populations, and health plans. It also evaluates characteristics of members who did versus did not enroll in the study, those who did versus did not engage in the program at different levels, and those who did versus did not complete the 12-month follow-up questionnaire.

#### Enrollment Rate

For members who received personal letters of invitation, the numerator of the enrollment rate is the number who enrolled in the study. The denominator is the total number of letters mailed minus the number of undeliverable letters. For members recruited through newsletters, the enrollment rate is the number who enrolled divided by the estimated total number of adult members estimated to be eligible, adjusted for the obesity rate in that state using Behavioral Risk Factor Survey data.

#### Levels of Program Engagement

Two measures of program engagement were calculated. To meet the criteria for initial engagement, participants needed to have viewed the initial electronic guide(s) relevant to their intervention. For example, participants in the Balance + Nourish intervention needed to have viewed the guides for both Balance and Nourish. The measure of ongoing engagement required, in addition, that participants view at least the initial follow-up electronic “newsletter” relevant to their intervention. These definitions were used because it was not possible in this study, given the data available, to examine engagement in more detail using continuous measures of program use.

#### 12-month Retention Rate

The numerator of the retention rate is the number of participants who completed a 12-month follow-up survey, via either email or regular mail. The denominator is the total number of participants who enrolled.

#### Characteristics of Enrollees Versus Nonenrollees

Among the members who were sent letters of invitation, information is available in health plan records about both those who enrolled and those who did not. In order to protect the confidentiality of those who did not enroll, we obtained group-level de-identified aggregate data for enrollees and nonenrollees; thus, individual-level or multivariate analyses are not possible. Similar group data were also obtained for members who attempted to enroll in the study but did not meet eligibility criteria. Characteristics used to compare these groups included age distribution (10-year age bands), gender, age × gender distribution, smoking status (yes/no), BMI distribution, and proportions with certain specific medical conditions, such as diabetes.

To evaluate the potential influence of health status and prevalence of chronic conditions that could influence participation, we employed a pharmacy-based risk adjustment system called RxRisk to identify comorbidities across the cohort of members contacted. The RxRisk system is a revised and expanded version of the original chronic disease score (CDS) risk assessment instrument [17,18]. RxRisk is a clinically validated algorithm that estimates expected medical care cost for the patient for the next year based on chronic disease categories and prescription drug fills. Both a continuous variable, which we refer to as RxRisk, along with dichotomous variables associated with various comorbidities were created using pharmacy dispensings in the 12 months prior to the recruitment period. Given the non-normal distribution of the RxRisk score, we dichotomized the score at the median.

#### Characteristics of Participants With Respect to Program Engagement and Completion of the 12-Month Survey

Baseline measures on which participants were compared included demographic factors (age, gender, race/ethnicity), medical characteristics (BMI, presence of diabetes or CAD), and psychosocial factors (baseline motivation and self-efficacy for coping with stress). Motivation was measured by an item that asked respondents how motivated they currently were to manage their weight (on a scale of 0 to 10, 10 = highest motivation). Self-efficacy was measured with an item that asked respondents to rate their confidence in being able to manage their weight when stressed (1 = “not confident” and 5 = “extremely confident”).

### Analyses

The chi-square statistic was used to determine the significance of differences on various characteristics between members who participated in the study and those who chose not to participate. Because the data for these enrollment analyses were de-identified and aggregated, we used the chi-square statistic rather than a multivariate statistic that would require individual-level data. Among participants, multiple logistic regression was used to identify predictors of the level of program engagement and retention at 12 months. After examining independent variables and adjusting for any non-normality, associations between independent variables were examined to avoid multicollinearity and compromising the stability of the models. Due to strong associations between region and race/ethnicity, separate analyses were conducted, one with each of the associated independent variables. Additionally, diabetes was strongly associated with region, so diabetes and region were not used in the same regression model.

**Figure 1:**
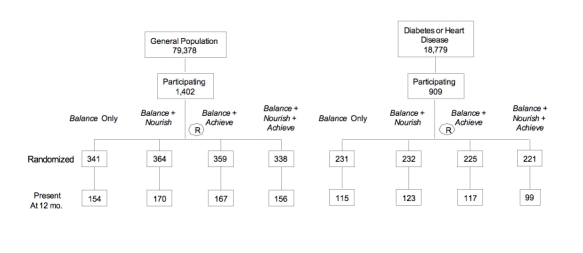
CONSORT diagram of participant retention

## Results

A total of 2311 members enrolled, 909 of which had a chronic illness (see Figure 1, showing recruitment streams and attrition). Overall, 54% of participants were 60 years of age or older, 45% had a BMI of 35 or higher, over 40% had hypertension, 14% had diabetes, and 12% had CAD. Table 1 shows that outreach letters to members with a chronic illness elicited a higher enrollment rate (overall approximately 5%) than did similar letters to general members (2.4%). Among those in the general membership (without known disease), the direct experimental comparison revealed that those sent a personal letter participated at a higher rate (2.4%) than those receiving the newsletter announcement (1.7%).

**Table 1:** Participation rate among those sent personal letters

	**Health Plan**		
**Population**	**HMO 1**	**HMO 2**	**HMO 3**
Diabetes registry members	6.6% (97/1477)^*^	4.8% (219/4606)	0.7% (15/2054)
CAD registry members	–	4.6% (194/4220)	2.6% (92/3496)
Hyperlipidemia registry members	10.0% (292/2926)	–	–
General population in specific geographic region	2.4% (59/2432)	–	–

^*^number of enrollees / number of personal letters of invitation sent.

We also found differences in enrollment rates across the three HMO settings. The HMO having the lowest enrollment rate also had participated in a similar, widely advertised, Internet-based weight loss project previously and had a higher prevalence of African Americans than did the other two HMOs. The other health plans had not participated in the prior study, so it is difficult to draw conclusions about why these differences occurred. Table 2 summarizes results from analyses on demographic and medical characteristics of those members who enrolled and those who declined. Those who enrolled were younger, were more likely to be nonsmokers and female, and were more likely to have lower RxRisk scores (less disease risk or burden, *P* < .001).

**Table 2:** Characteristics of enrollees and decliners

Characteristic	Enrollees (%)	Decliners (%)	*P* value
**Age**			
< 60 years	53.5	40.0	< .001
> 60 years	46.5	60.0
**Gender**			
Male	46.7	58.1	< .001
Female	53.3	41.9
**Smoking Status**			
Current smoker	5.7	12.2	< .001
Nonsmoker	94.3	87.8
**RxRisk (estimated medical costs)**			
< US $3000	55.6	44.3	< .001
> US $3000	44.4	55.7

### Engagement

Program engagement varied widely by intervention (Table 3). The vast majority of participants viewed the initial Balance guide, and approximately half met the criteria for ongoing Balance engagement. As can be seen, substantially fewer participants met criteria for engagement in the other interventions. This might be expected since these criteria were more stringent and required more of participants (eg, those in the Balance + Achieve intervention had to meet requirements for both the Balance and Achieve programs). Both initial and ongoing engagement rates were especially low for the Nourish intervention, likely because this program was only initiated after the initial intervention period for Balance users.

**Table 3:** Percent of patients achieving different levels of program engagement, by intervention

Intervention	Initial Engagement^*^ (%)	Ongoing Engagement^†^ (%)
Balance only (n = 572)	90.9	49.0
Balance + Achieve (n = 584)	62.2	25.3
Balance + Nourish (n = 596)	19.1	8.1
Balance + Achieve + Nourish (n = 559)	13.4	5.7

^*^Initial Engagement = Viewed initial electronic guides appropriate to that intervention

^†^ Ongoing Engagement = Initial engagement plus viewed at least initial electronic follow-up newsletters appropriate to intervention (or for Achieve, set at least initial goal)

As can be seen in Table 4, the intervention was the primary factor associated with initial engagement (the reference category for treatment effects was participants not randomized to a given intervention). All of the significant treatment effects and interactions with treatment indicated that the basic Balance intervention produced substantially higher initial engagement rates than the others. The only other variable to reach significance was gender, with females being significantly more likely than males to view initial guides (*P* < .001).

Analyses of ongoing engagement revealed a number of significant associations (lower portion of Table 4). All of the factors significant for initial engagement were also significant predictors of ongoing engagement. In addition, older participants (*P* < .001) and those with higher baseline motivation levels (*P* = .04) were more likely to demonstrate ongoing engagement. In contrast, African Americans (*P* = .03) and those with higher baseline self-efficacy scores (*P* = .003) were less likely to be engaged with their program on an ongoing basis.

**Table 4:** Results of logistic regression to predict engagement

Factor	Odds Ratio (CI)	Beta	SE	*P* value
**Predictors of Initial Engagement (N = 2276)**				
Nourish (vs non)	0.02 (0.02-0.03)	−3.78	0.18	< .001
Achieve (vs non)	0.17 (0.12-0.23)	−1.79	0.17	< .001
Nourish × Achieve (Balance only)	3.97 (2.49-6.32)	1.38	0.24	< .001
Baseline BMI	0.99 (0.97-1.00)	−0.02	0.01	.10
Age	1.01 (1.00-1.02)	0.01	0.01	.27
Female	1.68 (1.27-2.22)	0.52	0.14	< .001
Ethnicity (see below)				.10
Diabetes diagnosis (vs non)	0.94 (0.73-1.20)	−0.07	0.13	.60
CAD diagnosis (vs non)	1.16 (0.83-1.60)	0.14	0.17	.39
Self-efficacy	0.95 (0.85-1.06)	−0.06	0.06	.32
Motivation	1.03 (0.97-1.10)	0.03	0.03	.27

**Predictors of Ongoing Engagement (N = 2276)**				
Nourish (vs non)	0.09 (0.06-0.12)	−2.46	0.18	< .001
Achieve (vs non)	0.34 (0.27-0.44)	−1.07	0.13	< .001
Nourish × Achieve (Balance only)	2.04 (1.19-3.48)	0.71	0.27	.009
Baseline BMI	0.99 (0.97-1.01)	−0.01	0.01	.44
Age	1.02 (1.01-1.03)	0.02	0.01	.001
Female	1.50 (1.12-2.01)	0.41	0.15	.006
Ethnicity				.04
White (vs non)	1.18 (0.92-1.51)	0.16	0.13	.20
Black / African American (vs non)	0.68 (0.47-0.97)	−0.39	0.18	.03
Hispanic/Latino	0.88 (0.56-1.38)	−0.13	0.23	.57
Diabetes diagnosis (vs non)	0.97 (0.76-1.25)	−0.03	0.13	.81
CAD diagnosis (vs non)	1.01 (0.73-1.40)	0.01	0.17	.97
Self-efficacy	0.84 (0.75-0.95)	−0.17	0.06	.003
Motivation	1.07 (1.00-1.14)	0.07	0.03	.04

### Retention

Approximately 48% of initial participants provided information at 12-month follow-up through either online or mailed surveys. Logistic regression analyses on characteristics of respondents and nonrespondents to this follow-up revealed a few significant factors (Table 5). Younger enrollees and those who had higher baseline levels of self-efficacy were less likely to participate in the follow-up. However, there were no significant effects of intervention type, BMI, gender, baseline motivation level, ethnicity, or presence or absence of either diabetes or CAD.

**Table 5:** Results of the multiple regression to predict retention at 12 months

Factor	Odds Ratio (CI)	Beta	SE	*P* value
Nourish (vs non)	1.11 (0.86-1.37)	0.08	0.12	.50
Achieve (vs non)	1.08 (0.83-1.33)	0.05	0.12	.67
Nourish × Achieve (vs non)	0.87 (0.59-1.15)	−0.19	0.17	.26
Baseline BMI	0.99 (0.98-1.01)	−0.01	0.01	.22
Age	1.01 (1.00-1.02)	0.01	0.004	< .001
Ethnicity^*^	1.00-1.19	−0.05 to 0.17	0.09-0.16	.32
Gender	0.94 (0.71-1.08)	−0.13	0.11	.23
Baseline self-efficacy	0.92 (0.84-0.99)	−0.10	0.04	.03
Baseline motivation	1.01 (0.96-1.06)	0.01	0.02	.75
Diabetes diagnosis (vs non)	0.99 (0.81-1.18)	−0.02	0.10	.82
CAD diagnosis (vs non)	0.87 (0.66-1.09)	−0.17	0.13	.19

^*^Ethnicity involved three separate contrasts: Hispanic vs Other, African American vs Other, and Non-Hispanic White vs Other.

## Discussion

Many HMO members are willing to participate in Internet-based weight management programs. Although the overall participation rate was not high in an absolute sense, eHealth programs may be an efficient way of delivering health promotion services to a large number of members. This would especially be so if these programs attract representative or high-risk participants. Representativeness analyses are essential to evaluate the public health impact of eHealth programs [5]. Our results were mixed on this issue: although we did attract many older patients with chronic illness, the enrollment analyses suggest that in general this program did not attract those at highest risk (smokers, older adults, etc) at the same rate as those at lower risk. However, among members in HMO 1 receiving letters of invitation, patients with CAD or diabetes were substantially more likely to participate than members without known chronic illness (10% and 7%, respectively, vs 2.4%).

Congruent with other computer-mediated programs, it appears that sending personal letters from health professionals is an effective method of enhancing enrollment rate [19-21]. Newsletter articles and flyers, in contrast, are a low-cost alternative but may recruit a considerably smaller proportion of the target audience. Although our evaluations of the type of recruitment and disease status were not randomized and were not orthogonal comparisons (with the exception of the comparisons within HMO 1), the results are fairly clear that personalized mailings increase enrollment rates, and members with a diagnosed chronic disease are more likely to participate than are other members.

Our engagement analyses revealed that adding components to a basic Internet-based intervention program can create adherence challenges. Although almost all participants viewed the initial Balance materials, far fewer viewed the other electronic guides in the Achieve and Nourish interventions. Intervention assignments were the factors most strongly associated with both initial and ongoing engagement; but, in addition, females were more likely to become and remain engaged than males. Additional demographic and motivational factors also predicted ongoing engagement (but not initial engagement). From an adherence perspective, it also appeared more successful to introduce additional treatment components during initial weight management stages (as in Achieve) than to wait until later (as in Nourish). Future studies should evaluate the use of the dichotomous engagement criteria used in this study compared to more sophisticated, automated engagement measures, such as patterns of log-ins over time.

As in many eHealth studies, there was substantial attrition by the time of the 12-month follow-up. Our attrition analyses suggest that those who declined to participate in the follow-up were generally representative of those who continued participation, the primary exceptions being baseline level of self-efficacy and age. Other recent eHealth research has found that dropping out of assessment is different from dropping out of intervention, and that those who drop out of eHealth programs may benefit just as much as those who do not [15], in contrast to typical findings in clinician- or educator-delivered programs. In terms of health disparities and health impact, it was encouraging that those who were older, weighed more, had diagnosed disease, and were members of racial/ethnic minority groups were equally or more likely to participate in follow-up assessments.

This study had both strengths and limitations. Strengths include the study of an eHealth program that proved efficacious in prior research [1]; analyses of a large-scale implementation of this program in three different “real-world” health care settings; the relatively comprehensive analyses of characteristics and the representativeness of those who enrolled versus declined, made possible by the electronic health record systems of the participating organizations; the inclusion of reach, engagement, and retention analyses; and the study of different recruitment methods. Limitations include the inability to conduct multivariable analyses of reach due to the Health Insurance Portability and Accountability Act (HIPAA) and privacy issues, the absence of information on some important factors such as health literacy, and the confounding of some issues such as diagnosed disease status and recruitment method.

Future research should compare the reach of Internet-based and other modalities of health promotion and investigate methods to enhance ongoing engagement and retention, which may be particular challenges for eHealth programs.

## Multimedia Appendix

Representativeness in eHealth Programs: Factors Related to Recruitment, Participation, and Retention - Poster (ppt)
	
						

## Abbreviations

BMI: body mass index
CAD: coronary artery disease
HMO: health maintenance organization
