# Supplementary material for: Reach, Engagement, and Retention in an Internet-Based Weight Loss Program in a Multi-Site Randomized Controlled Trial
Source: J Med Internet Res. 2007 May 9;9(2):e11. doi: 10.2196/jmir.9.2.e11 (PMC1874718; doi:10.2196/jmir.9.2.e11)
Supplement: Supplementary file 1 [file jmir_v9i2e11_app1.ppt]

## Slide 1
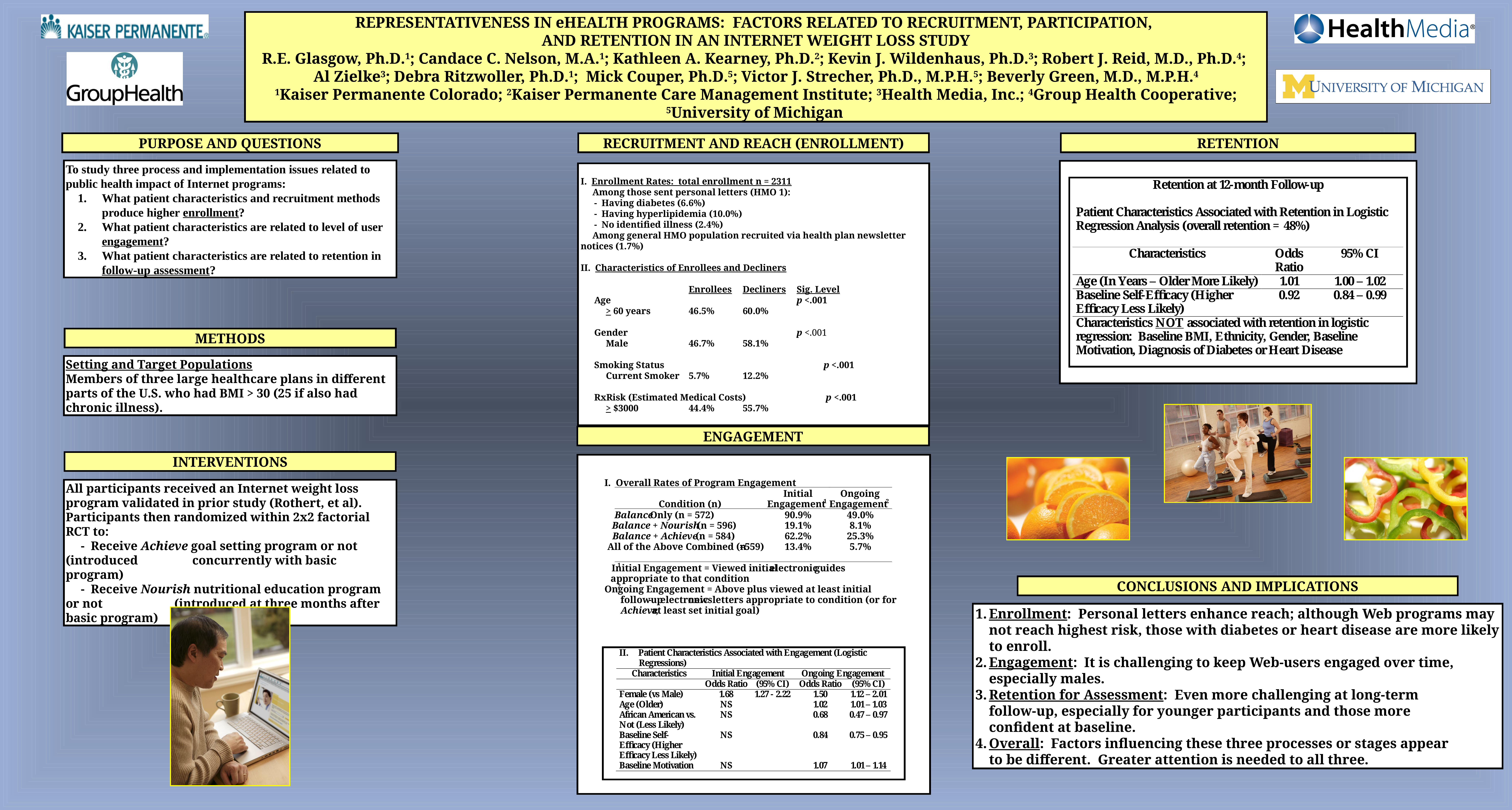

REPRESENTATIVENESS IN eHEALTH PROGRAMS: FACTORS RELATED TO RECRUITMENT, PARTICIPATION,
AND RETENTION IN AN INTERNET WEIGHT LOSS STUDY
R.E. Glasgow, Ph.D.1; Candace C. Nelson, M.A.1; Kathleen A. Kearney, Ph.D.2; Kevin J. Wildenhaus, Ph.D.3; Robert J. Reid, M.D., Ph.D.4;
Al Zielke3; Debra Ritzwoller, Ph.D.1; Mick Couper, Ph.D.5; Victor J. Strecher, Ph.D., M.P.H.5; Beverly Green, M.D., M.P.H.4
1Kaiser Permanente Colorado; 2Kaiser Permanente Care Management Institute; 3Health Media, Inc.; 4Group Health Cooperative; 5University of Michigan
PURPOSE AND QUESTIONS
To study three process and implementation issues related to public health impact of Internet programs:
 1. 	What patient characteristics and recruitment methods 			produce higher enrollment?
 2. 	What patient characteristics are related to level of user 			engagement?
 3. 	What patient characteristics are related to retention in 			follow-up assessment?
RECRUITMENT AND REACH (ENROLLMENT)
I. Enrollment Rates: total enrollment n = 2311
 Among those sent personal letters (HMO 1):
	- Having diabetes (6.6%)
	- Having hyperlipidemia (10.0%)
	- No identified illness (2.4%)
 Among general HMO population recruited via health plan newsletter notices (1.7%)
II. Characteristics of Enrollees and Decliners
							Enrollees	Decliners	Sig. Level
	Age 							 		p <.001
		> 60 years		46.5%		60.0%
	Gender								p <.001
	 	Male				46.7%		58.1%
	Smoking Status						p <.001
		Current Smoker	5.7%		12.2%
	RxRisk (Estimated Medical Costs)			 p <.001
		> $3000		44.4%		55.7%
RETENTION
METHODS
Setting and Target Populations
Members of three large healthcare plans in different parts of the U.S. who had BMI > 30 (25 if also had chronic illness).
ENGAGEMENT
I. Overall Rates of Program Engagement
Initial
Ongoing
1
2
Condition (n)
Engagement
Engagement
Balance
 Only (n = 572)
90.9%
49.0%
Balance + Nourish
 (n = 596)
19.1%
8.1%
Balance + Achieve
 (n = 584)
62.2%
25.3%
All of the Above Combined (n
=559)
13.4%
5.7%
1
Initial Engagement = Viewed initial
electronic
guides
appropriate to that condition
2
Ongoing Engagement = Above plus viewed at least initial
follow
-
up
electronic
newsletters appropriate to condition (or for
Achieve
at least set initial goal)
,
INTERVENTIONS
All participants received an Internet weight loss program validated in prior study (Rothert, et al). Participants then randomized within 2x2 factorial RCT to:
	- Receive Achieve goal setting program or not (introduced 			concurrently with basic program)
	- Receive Nourish nutritional education program or not 				(introduced at three months after basic program)
CONCLUSIONS AND IMPLICATIONS
Enrollment: Personal letters enhance reach; although Web programs may
not reach highest risk, those with diabetes or heart disease are more likely
to enroll.
Engagement: It is challenging to keep Web-users engaged over time,
especially males.
Retention for Assessment: Even more challenging at long-term
follow-up, especially for younger participants and those more
confident at baseline.
Overall: Factors influencing these three processes or stages appear
to be different. Greater attention is needed to all three.
